# Integrating machine learning and multi-omics analysis to identify and validate key genes associated with periodontitis

**DOI:** 10.2340/aos.v85.46662

**Published:** 2026-08-19

**Authors:** Yierfan Nuermaimaiti, Reyila Jureti, Aierpati Maimaiti, Yiming Li, Gulinuer Awuti

**Affiliations:** a Department of Periodontology, Xinjiang Medical University Affiliated First Hospital, Urumqi, Xinjiang, People’s Republic of China; b Department of Neurosurgery, Xinjiang Medical University Affiliated First Hospital, Urumqi, Xinjiang, People’s Republic of China

**Keywords:** Multi-omics, machine learning, Mendelian randomization, biomarker, periodontitis

## Abstract

**Aims:**

Early detection and intervention are crucial for effective management of periodontitis. Our study aims to identify diagnostic biomarkers for periodontitis by integrating single-cell RNA sequencing analysis, Mendelian randomization, and experimental validation.

**Methods:**

Gene Expression Omnibus (GEO) dataset GSE164241 was downloaded to analyze the cellular compositions and intercellular communications in periodontitis.

GEO datasets GSE10334, GSE16134, GSE23586, and GSE106090 were downloaded, and differential expression genes (DEGs) and functional enrichment were analyzed, followed by investigation of endothelial-fibroblast signaling pathways. Molecular subtypes were defined based on myeloid and fibroblast markers, and their immune characteristics were analyzed. A diagnostic model was built using 106 algorithm combinations from 11 machine learning methods (Lasso, Ridge, Enet, Stepglm, SVM, glmBoost, LDA, RandomForest, GBM, XGBoost, and NaiveBayes) to screen reliable biomarkers. Key genes were identified using Mendelian randomization and validated via reverse transcription and quantitative polymerase chain reaction (RT-qPCR).

**Results:**

This study identified four novel periodontitis subtypes using consensus clustering based on myeloid cell and fibroblast markers: fibroblast-dominant, myeloid-dominant, fibroblast/myeloid quiescent, and fibroblast/myeloid mixed. These four subtypes exhibited unique biological patterns, potentially leading to different disease progression. Patients in the fibroblast/myeloid mixed and myeloid-dominant groups, in particular, may have a stronger inflammatory microenvironment. In addition, we found that IGFBP4, IL1B, LAPTM5, PSAP, and SRGN were closely related to the occurrence and development of periodontitis through random forest (RF) analysis and Mendelian randomization. RT-qPCR validation in gingival tissues from 13 stage III/IV periodontitis patients and healthy controls confirmed significant differences in the expression of IGFBP4, IL1B, and LAPTM5 (*p* < 0.001).

**Conclusion:**

Our findings suggest that IGFBP4, IL1B, and LAPTM5 could be potential biomarkers for periodontitis, paving the way for future research on its pathogenesis, diagnosis, and treatment.

## Summary

### Background

Periodontitis, a chronic inflammatory disease causing irreversible bone loss and tooth exfoliation, lacks early diagnostic tools. Current methods rely on late-stage clinical signs, underscoring the need for molecular insights. Single-cell RNA sequencing (scRNA-seq) enables cellular-resolution analysis of disease mechanisms, including immune-stromal crosstalk and transcriptional heterogeneity, offering transformative potential for precision diagnostics and therapies.

### Added value of this study

We identify four molecular subtypes (fibroblast-dominant, myeloid-dominant, quiescent, and mixed) via integrative scRNA-seq and machine learning. Mixed/myeloid subtypes exhibit hyperinflammatory microenvironments, while Mendelian randomization and experimental validation (*p* < 0.001) implicate IGFBP4, IL1B, and LAPTM5 as causal drivers. These genes link to osteogenic remodeling (LAPTM5) and inflammasome activation (IL1B), establishing a molecular taxonomy bridging cellular heterogeneity to clinical phenotypes.

### Clinical implications

Our findings enable precision therapies: anti-inflammatory regimens for mixed/myeloid subtypes and regenerative strategies for fibroblast-dominant cases. IGFBP4/IL1B/LAPTM5 serve as early biomarkers and therapeutic targets, with IL1B inhibition and LAPTM5 modulation offering actionable strategies. Machine learning models enhance risk stratification, guiding proactive management. This work redefines periodontitis care through molecular-driven subtyping, advancing personalized prevention and treatment.

## Introduction

Periodontitis is an inflammatory destructive disease of the periodontal supporting tissues. Plaque-induced gingival inflammation triggers a severe immune response, destroying periodontal attachment and resulting in tooth loosening, displacement, and loss [1]. Moreover, periodontitis is the most common cause of tooth loss in adults [2]. The consequences of periodontitis include functional impairment of mastication, nutritional deficiencies, and even psychological issues. In the last two decades, periodontitis has risen to become the sixth most prevalent chronic noncommunicable disease worldwide [3]. This has led to a 57.3% increase in the global burden, a figure that continues to climb, affecting the oral health and quality of life of millions [4]. At present, the diagnosis of periodontitis predominantly relies on a combination of clinical examination, radiographic assessment, and patient medical history [5]. However, these conventional diagnostic methods exhibit inherent limitations, which impede the early identification and definitive diagnosis of periodontitis. Periodontal tissues are now understood to be a dynamic organ with regenerative potential, as recent research has shown. The critical interplay between cells responsible for tissue resorption, formation, and immune responses is primarily regulated by paracrine, endocrine, juxtacrine, and autocrine signaling pathways, mirroring the mechanisms in other organs [6]. It is therefore critical to conduct thorough research into the molecular mechanisms driving the development and progression of periodontitis to uncover novel biomarkers and therapeutic targets.

The rapid development of single-cell RNA sequencing (scRNA-seq) has revolutionized our ability to analyze complex biological systems [7]. This technology allows for the capture of gene expression profiles at the single-cell level, enabling detailed characterization and functional analysis of diverse cell populations [8]. Periodontitis is a chronic inflammatory disease that results from the complex interplay of multiple factors, cell types, and signaling pathways [1]. Using single-cell RNA sequencing technology, we can identify characteristic gene expression patterns in different cell types within periodontal tissues, revealing their specific roles in disease progression [9]. The investigation of innate immunity and fibroblast remodeling is of considerable importance in the study of periodontitis [10]. However, research in this area is relatively scarce. The innate immune system represents a critical component of the early defense response to periodontitis, and fibroblasts are essential for the structural maintenance and repair of periodontal tissues [11]. Single-cell RNA sequencing provides a powerful tool to delve into the specific functions and interactions of these cells in periodontitis, uncovering key regulatory mechanisms driving disease development.

Our study reveals novel insights into the molecular mechanisms of periodontitis. By integrating multi-omics data with machine learning and Mendelian randomization (MR) analyses, we identified novel disease subtypes, associated genes, and key regulatory pathways. These findings were validated using reverse transcription and quantitative polymerase chain reaction (RT-qPCR) in gingival tissue samples from a cohort of periodontitis patients and healthy controls (*n* = 13 and *n* = 15, respectively). This investigation presents potentially efficacious novel approaches for the targeted treatment of periodontitis.

## Methods

### Raw data collection and processing

To elucidate the complex periodontal microenvironment at the cellular level, we obtained human single-cell transcriptomic data (scRNA-seq) from GEO (GSE164241; https://www.ncbi.nlm.nih.gov/geo/), comprising oral mucosal tissue from eight periodontitis patients. For identifying key diagnostic biomarkers, we further downloaded whole-transcriptome datasets (GSE10334, GSE16134, GSE23586, and GSE106090). To mitigate batch effects in the larger GSE10334 and GSE16134 datasets, we employed the ‘Combat’ algorithm (sva R package) before molecular subtype identification.

### Single-cell-based biomarker identification

To characterize cell populations, we performed single-cell RNA sequencing data analysis using the Seurat R package. Rigorous quality control steps ensured data reliability. Batch effects were corrected using RunHarmony, facilitating downstream analyses. Dimensionality reduction and clustering identified distinct cell populations, which were manually annotated based on known marker genes. Functional enrichment analysis (GO, KEGG, Reactome) revealed the key biological pathways and functions enriched in each cell population, providing insights into the cellular heterogeneity within the periodontal microenvironment. Word cloud visualization further summarized the key biological functions of each cell cluster [12].

### Ligand–receptor interaction analysis

Cell–cell communication, fundamental to intercellular information exchange and diverse biological processes, was quantified using the ‘CellChat’ R package. Aligning with pattern recognition and multitask learning principles, we identified and visualized dominant sender and receiver cells in a 2D space. We then analyzed and visualized ligand–receptor mediated interactions among the 14 cell types, focusing on endothelial and fibroblast interactions with other cell types and their key signaling pathways.

### Identifying molecular subtype

Four molecular subtypes were identified by consensus clustering (ConsensusClusterPlus; reps = 100, pItem = 0.8, pFeature = 1) of myeloid and fibroblast marker genes. Subtypes were defined based on median expression levels of these markers as static (myeloid < 0, fibroblast < 0), myeloid-dominant (myeloid > 0, fibroblast < 0), fibroblast-dominant (myeloid < 0, fibroblast > 0), and mixed (myeloid > 0, fibroblast > 0) [13]. The biological uniqueness of each subtype was investigated by comparing their immune features. Differences in HLA expression between subtypes were assessed using nonparametric statistical tests: the Kruskal–Wallis test for multigroup comparisons and the Wilcoxon test for pairwise comparisons. Immune cell infiltration was further investigated using three deconvolution algorithms: MCPcounter, CIBERSORT, and ssGSEA [14, 15].

### Identifying hub genes

To enhance the robustness of biomarker identification in periodontitis, we employed an ensemble approach integrating 11 diverse machine learning algorithms (Lasso, Ridge, Elastic Net, Stepwise GLM, SVM, GLM Boost, LDA, Random Forest, GBM, XGBoost, and Naive Bayes). This ensemble model was assessed by calculating the AUC on a held-out validation dataset to evaluate its diagnostic accuracy [16].

### Mendelian randomization analysis of key genes

A summary-data-based Mendelian randomization (SMR) workflow was implemented to identify genetic variants associated with target genes. This involved: downloading expression quantitative trait locus (eQTL) data from GTEx v8, selecting tissues based on their correlation with target gene expression and processing these data using the SMR tool (downloaded from http://cnsgenomics.com/software/smr/), ensuring compatibility with downstream analyses [17].

To ensure data quality and consistency, periodontitis outcome data were retrieved from the IEU OpenGWAS database (https://gwas.mrcieu.ac.uk/) using the finn-b-K11_PERIODON_ACUTE dataset. The download process involved accessing the IEU OpenGWAS website, locating the dataset, and downloading the relevant data. Downloaded data were carefully checked to ensure the inclusion of crucial information: single nucleotide polymorphism (SNP) ID, effect allele, effect size (beta), standard error (SE), and *p*-value. These data underwent a formatting process for compatibility with MR analysis.

Rigorous quality control was applied to the integrated eQTL and outcome data. After matching the datasets by SNP ID to ensure each SNP had corresponding effect size and statistical information in both, SNPs were excluded if they had: a minor allele frequency (MAF) < 0.01, a Hardy–Weinberg equilibrium *p*-value < 1e-6, or a missingness rate > 5%.

The integrated data underwent MR analysis using the inverse variance weighted (IVW) method. This involved inputting the data into a suitable MR analysis software package and employing the IVW method to estimate the causal effect of gene expression on the outcome of interest. The IVW method synthesizes the effects of multiple SNPs through a weighted average, providing a robust overall effect size estimate and enabling identification of hub genes within the set of key genes.

### Patient recruitment and ethics

This study utilized a case–control design, recruiting 13 systemically healthy periodontitis patients (case group) and 15 systemically and periodontally healthy volunteers (control group) from the Periodontology Department of Xinjiang Medical University’s First Affiliated Hospital/Affiliated Stomatological Hospital between February and May 2024. Healthy controls were included if they met the following criteria: (1) age between 18 and 35 years; (2) demonstrated good oral hygiene; (3) showed no clinical signs of periodontitis, including a probing depth (PD) of ≤ 3 mm, bleeding on probing (BOP) in less than 10% of sites, and no attachment loss (AL) [18, 19]; and (4) had undergone extraction of at least one tooth for orthodontic treatment [20].

Periodontitis diagnosis followed the 2018 International Classification of Periodontal and Peri-Implant Diseases [21]. Staging was determined by the number of teeth lost due to periodontitis: stage III was defined as ≤ 4 missing teeth and stage IV as ≥ 5 missing teeth. Case group inclusion criteria were: (1) Stage III or IV periodontitis 2018 International Classification of Periodontal and Peri-Implant Diseases [22] and (2) at least one hopeless tooth requiring extraction. Both case and control groups excluded participants with: (1) any systemic disease; (2) antibiotic use within the past 3 months; (3) pregnancy, breastfeeding, or a history of oral contraceptive use (for female participants); (4) any periodontal treatment within the past 6 months; (5) a history of smoking; and (6) a history of orthodontic treatment or significant malocclusion.

This study adhered to the Declaration of Helsinki and received ethical approval from the Ethics Committee of the First Affiliated Hospital of Xinjiang Medical University (Urumqi, Xinjiang Uygur Autonomous Region, China). Gingival tissue samples were collected from 13 patients with stage III or IV periodontitis (during extraction of hopeless teeth) and 15 periodontally healthy volunteers (during orthodontic extractions). All participants provided informed consent prior to sample collection. Samples were stored in Eppendorf tubes at −80°C.

### qRT-PCR

Total RNA was extracted from gingival tissue samples obtained from both periodontitis patients and healthy controls using an RNA extraction kit (Servicebio, Wuhan, China), following the manufacturer’s recommended protocol. The concentration and purity of the isolated RNA were quantified using a Nanodrop 2000 spectrophotometer (Thermo Fisher Scientific). RT-qPCR were performed using a QuantStudio 3 Real-Time PCR System and ChamQ Universal SYBR qPCR Master Mix (Thermo Fisher Scientific). Relative gene expression levels were calculated using the comparative Ct method (2^–ΔΔCt, where ΔCt = Ct value of target gene – Ct value of reference gene; ΔΔCt = mean ΔCt of experimental group – mean ΔCt of control group), with glyceraldehyde-3-phosphate dehydrogenase (GAPDH) mRNA serving as the internal reference. The nucleotide sequences of the primers used in this study are listed in Supplementary Table 1.

### Data analysis

Statistical analysis was conducted using R software (version 4.4). Histological data, which were ordinal, were analyzed using the Kruskal–Wallis test. If a statistically significant difference was found (*p* < 0.05), post-hoc pairwise comparisons were performed using the Mann–Whitney U test. RT-qPCR data were assessed for normality and homogeneity of variance. Meeting these assumptions, a one-way analysis of variance (ANOVA) was employed to compare groups. A *p*-value < 0.001 was considered statistically significant.

## Results

### Identifying key communicating cells via single-cell annotation

**Figure 1:**
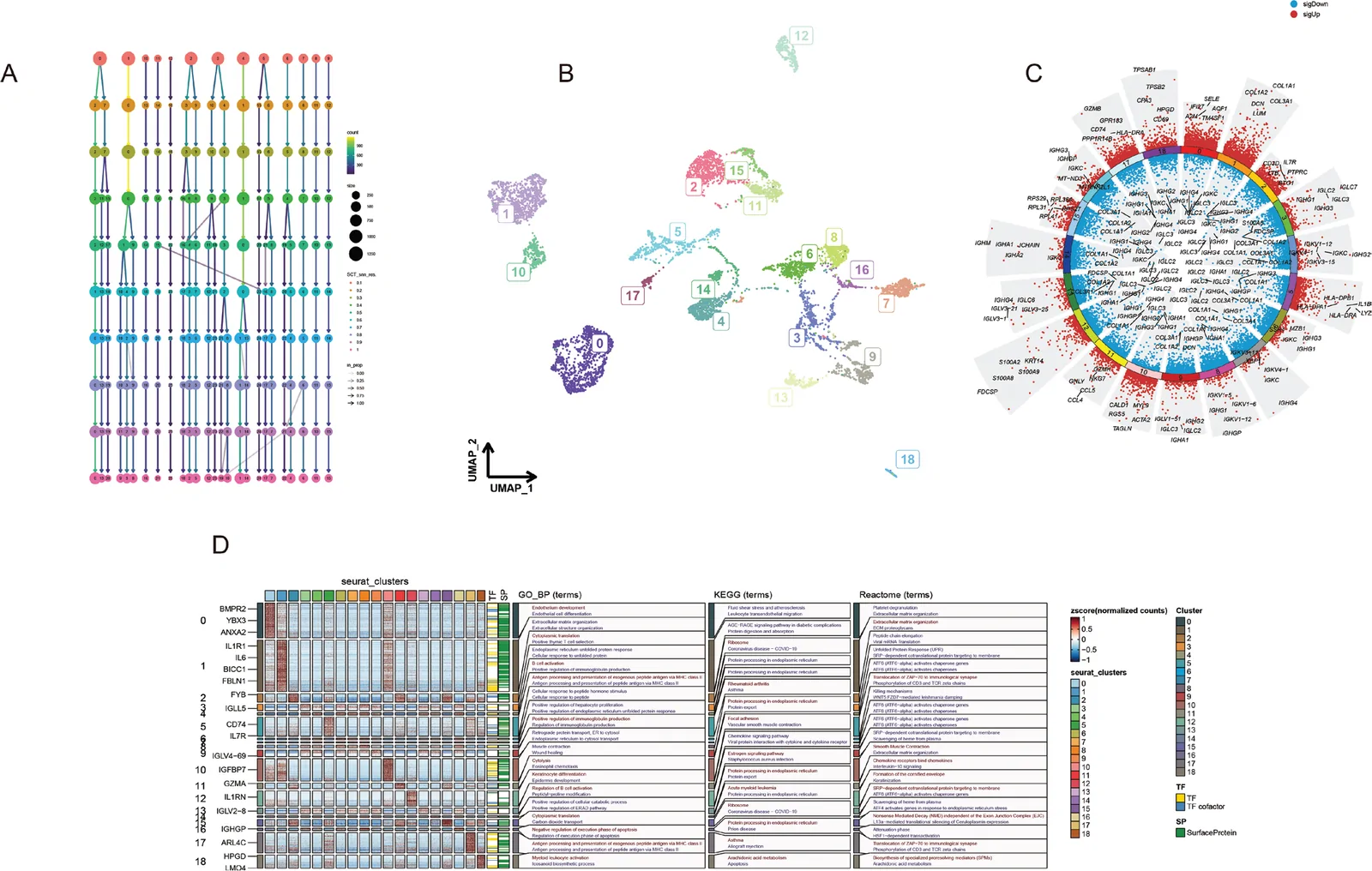
Profiles of cells in the microenvironment of periodontitis on the scRNA transcriptome level. (A) Clustering tree for the cells clustered using various resolution parameters, and the best resolution was 0.4. (B) The UMAP algorithm helped visualize 19 various cell clusters. (C) The Volcano function can be used to visualize marker genes in multiple clusters. (D) 13 types of cells were annotated as the main labels of the cluster. The proportion of each in the three samples was calculated. The expression landscape of specific annotation-based markers in cell clusters after annotation.

Nineteen distinct single-cell subpopulations (Figure 1A, B) were identified, each displaying a unique transcriptional profile (Figure 1C). Functional enrichment analysis of differentially expressed genes revealed distinct biological functions and lineage specificities for each subpopulation (Figure 1D).

**Figure 2:**
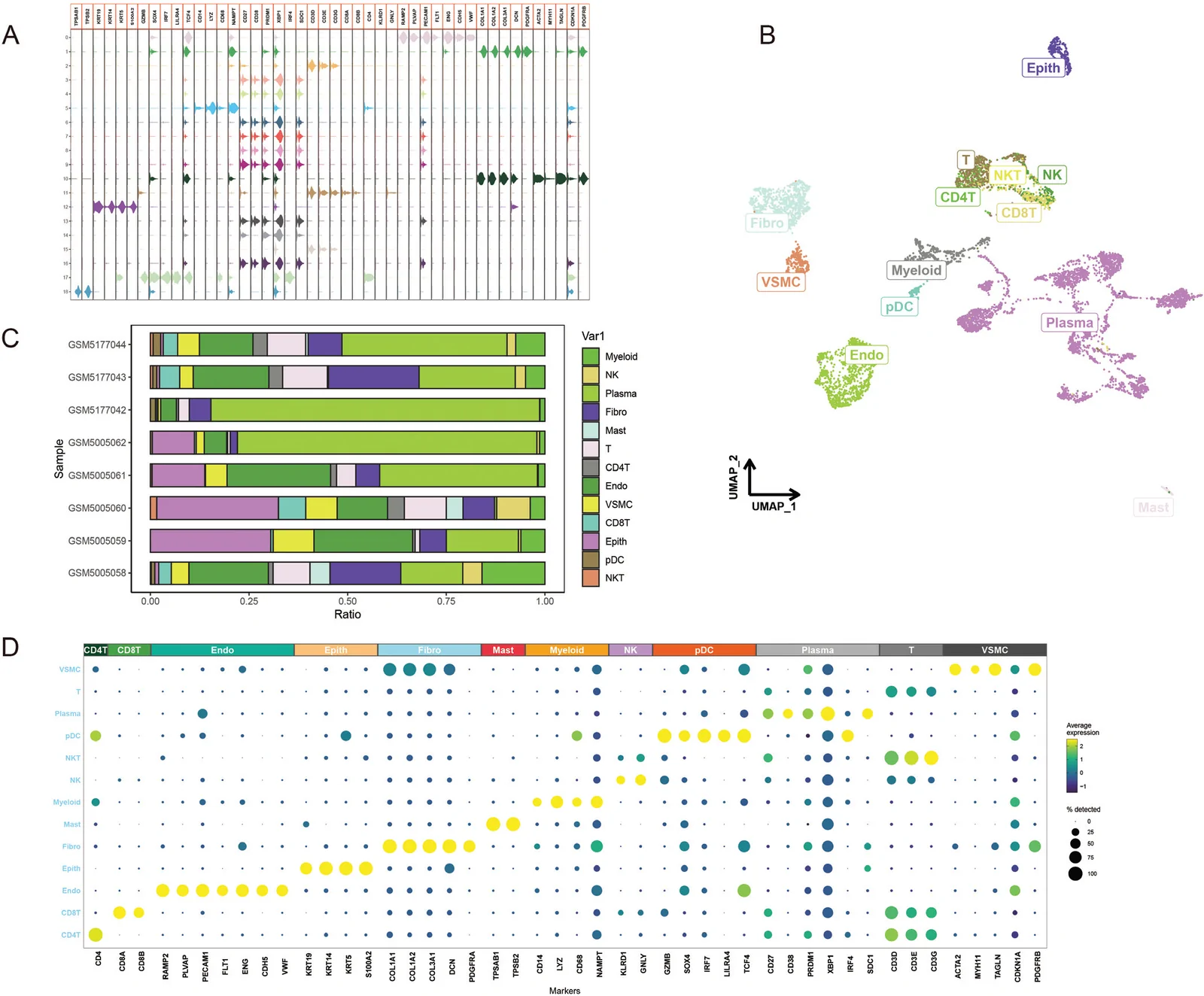
(A) Violin plots showing the expression distribution of cell type-specific marker genes across the identified clusters. (B) UMAP plot visualizing the clustering of cells into distinct groups based on their transcriptional profiles, with each cluster labeled by its corresponding cell type. (C) Bar plot illustrating the proportion of each annotated cell type in different samples. (D) Dot plot describing the expression landscape of marker genes across annotated clusters. The size of the dots indicates the percentage of cells expressing the respective marker gene, and the color represents the average expression level.

Based on the expression profiles of lineage-specific marker genes, the 19 single-cell clusters were classified into 13 distinct cell types (Figure 2A, B). The proportion of each cell type across eight samples was calculated (Figure 2C). Cell type annotation was based on the expression of lineage-specific marker genes (Figure 2D). Cell types were annotated based on marker gene expression: T cells (CD3D, CD3E, CD3G; CD8+ T cells additionally expressed CD8A or CD8B; CD4+ T cells expressed CD4; NKT cells expressed KLRD1 and GNLY); pDCs (GZMB, SOX4, IRF7, LILRA4, TCF4); endothelial cells (RAMP2, PLVAP, PECAM1, FLT1, ENG, CDH5, VWF); fibroblasts (COL1A1, COL1A2, COL3A1, DCN, PDGFRA); VSMCs (ACTA2, MYH11, TAGLN, CDKN1A, PDGFRB); myeloid cells (CD14, LYZ, CD68, NAMPT); plasma cells (GZMB, SOX4, IRF7, LILRA4, TCF4); epithelial cells (KRT19, KRT14, KRT5, S100A2); and mast cells (TPSAB1, TPSB2).

**Figure 3:**
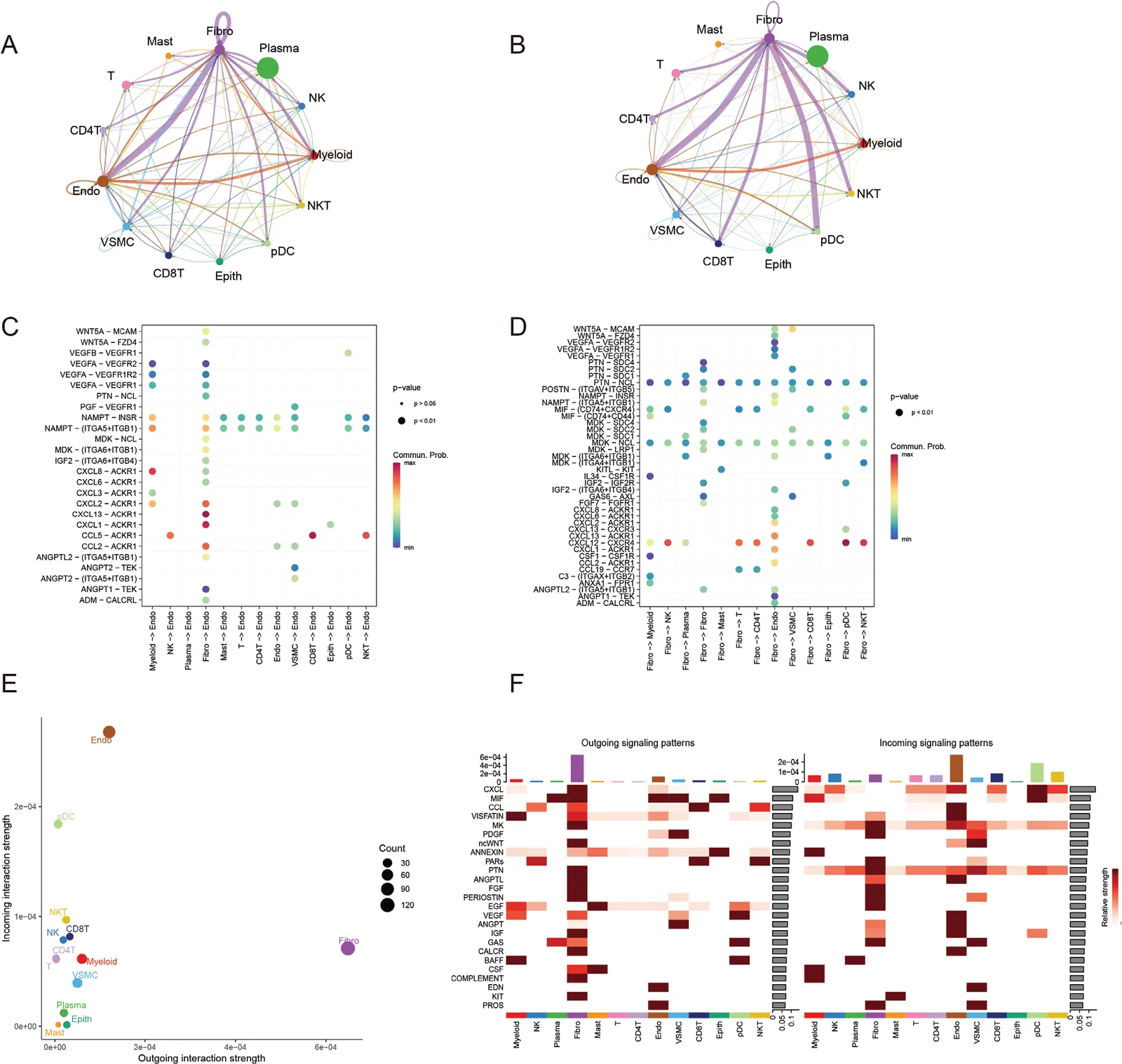
The landscape of cell–cell communication. (A, B) The circle diagrams show the interaction strength hand number between different cells. (C, D) Dot plots presenting ligand–receptor interactions between different cell types. The y-axis shows specific ligand–receptor pairs, while the x-axis represents interacting cell types. Dot size reflects the significance of interaction (*p*-value), and color intensity indicates the communication probability. (E) Scatter plot of incoming versus outgoing interaction strength for each cell type. Each point corresponds to a specific cell type, with bubble size representing the number of unique interactions. (F) Heatmaps of signaling patterns for each cell type.

Visualization of cell–cell communication within the periodontitis microenvironment revealed substantial variation in both the strength and frequency of interactions. This variation was observed both in the types of ligand–receptor pairs involved (differing between incoming and outgoing signals) and across different cell types. Significantly, endothelial cells received, and fibroblasts emitted, a large number of signals (Figure 3A, B). Chemokine-mediated signaling pathways were predominantly involved in these interactions (Figure 3C, E). Notably, CXCL and MIF signaling pathways played a key role, mediating numerous cell–cell communication events (Figure 3F).

### Identify molecular subtypes

Periodontitis is an inflammatory disease driven by a dense accumulation of immune cells within the affected tissues. This accumulation and subsequent activation of innate immune cells within the oral environment represent a critical transition phase between the initial innate immune response and the later development of an adaptive immune response [1]. The cellular composition at this stage is primarily comprised of innate immune cells, with dendritic cells (DCs), macrophages, and natural killer (NK) cells constituting the predominant cell populations [23].

The adaptive immune response is subsequently initiated by the presentation of antigens to T and B lymphocytes by antigen-presenting cells (APCs), including DCs and macrophages [22]. This results in a transition to a phase dominated by these adaptive immune cells.

**Figure 4:**
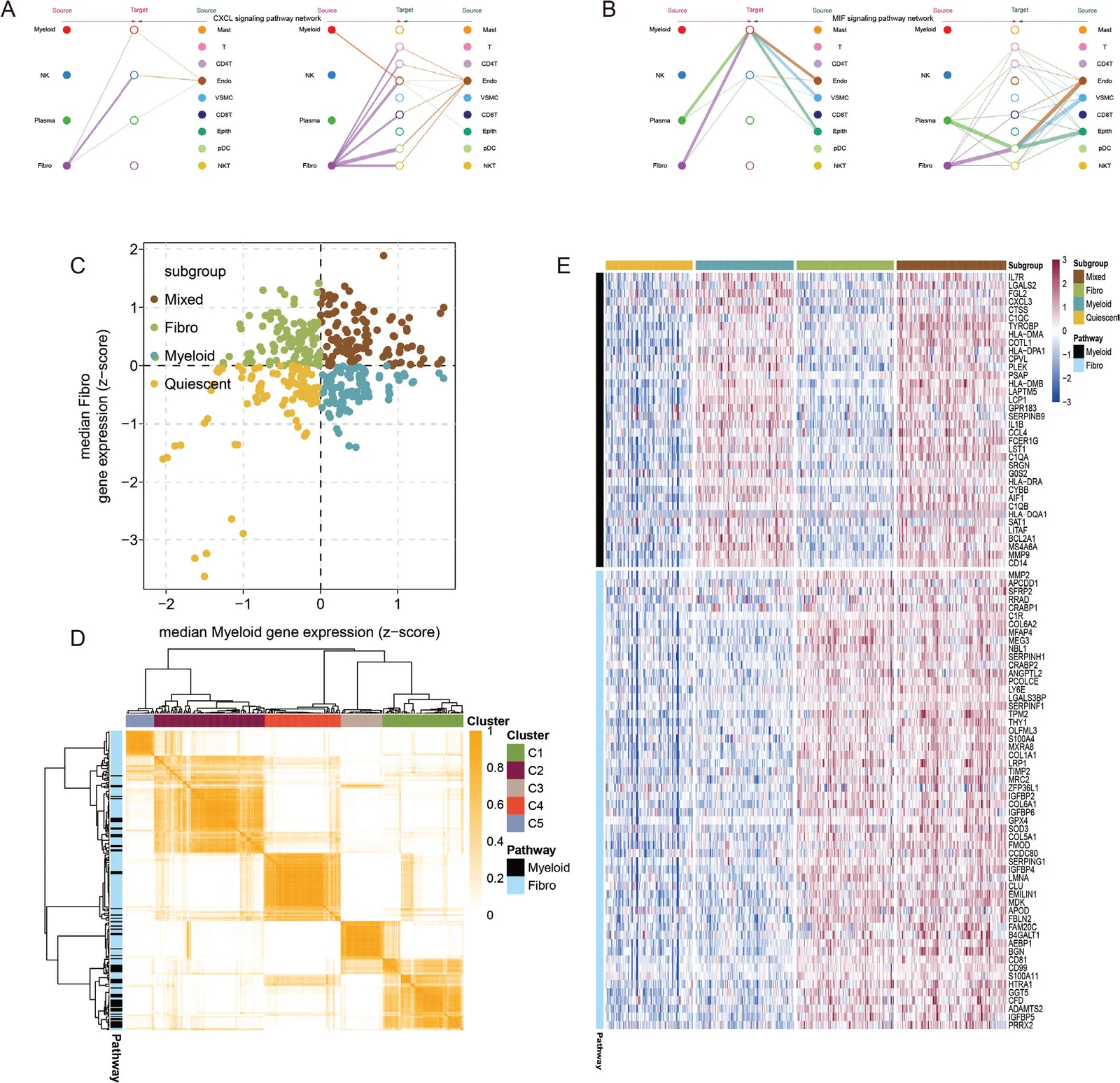
(A, B) Network diagrams representing the cell-to-cell communication within periodontal tissues. (A) Highlights the CXCL pathway, illustrating the source and target cells, with myeloid cells and fibroblasts serving as primary mediators. (B) Displays the TNF signaling pathway, further emphasizing the critical roles of myeloid cells and fibroblasts in periodontal disease pathogenesis. The size of the nodes reflects the intensity of signal strength, and the lines represent interactions between cell sources and targets. (C) Scatter plot depicting the median expression of fibroblast-specific (y-axis) and myeloid-specific (x-axis) feature genes across four novel periodontal subtypes: mixed, fibro, myeloid, and quiescent. Colored points represent individual samples clustered by consensus expression patterns. (D) Consensus clustering heatmap based on myeloid and fibroblast pathway-specific gene expression. Subclasses (C1–C4) are stratified by predominant pathway involvement, indicating the distinct cellular contributions to periodontal disease. (E) Heatmap showing the expression profiles of myeloid- and fibroblast-enriched feature genes across the four subtypes. Red and blue indicate high and low expression levels, respectively. Subtypes are color coded and labeled at the top of the heatmap.

To investigate the interplay between myeloid cells and fibroblasts in periodontitis, we analyzed their respective marker genes within a signaling pathway network (Figure 4A, B). Consensus clustering of myeloid and fibroblast markers revealed two major clusters: C4, enriched for fibroblast markers, and C1, enriched for myeloid markers (Figure 4C). Analysis of median expression levels of characteristic genes from these clusters identified four novel periodontitis subtypes: fibroblast-dominant, myeloid-dominant, fibroblast/myeloid-quiescent, and fibroblast/myeloid mixed. The expression patterns of C1 and C4 marker genes across these four subtypes are shown in Figure 4D, E.

**Figure 5:**
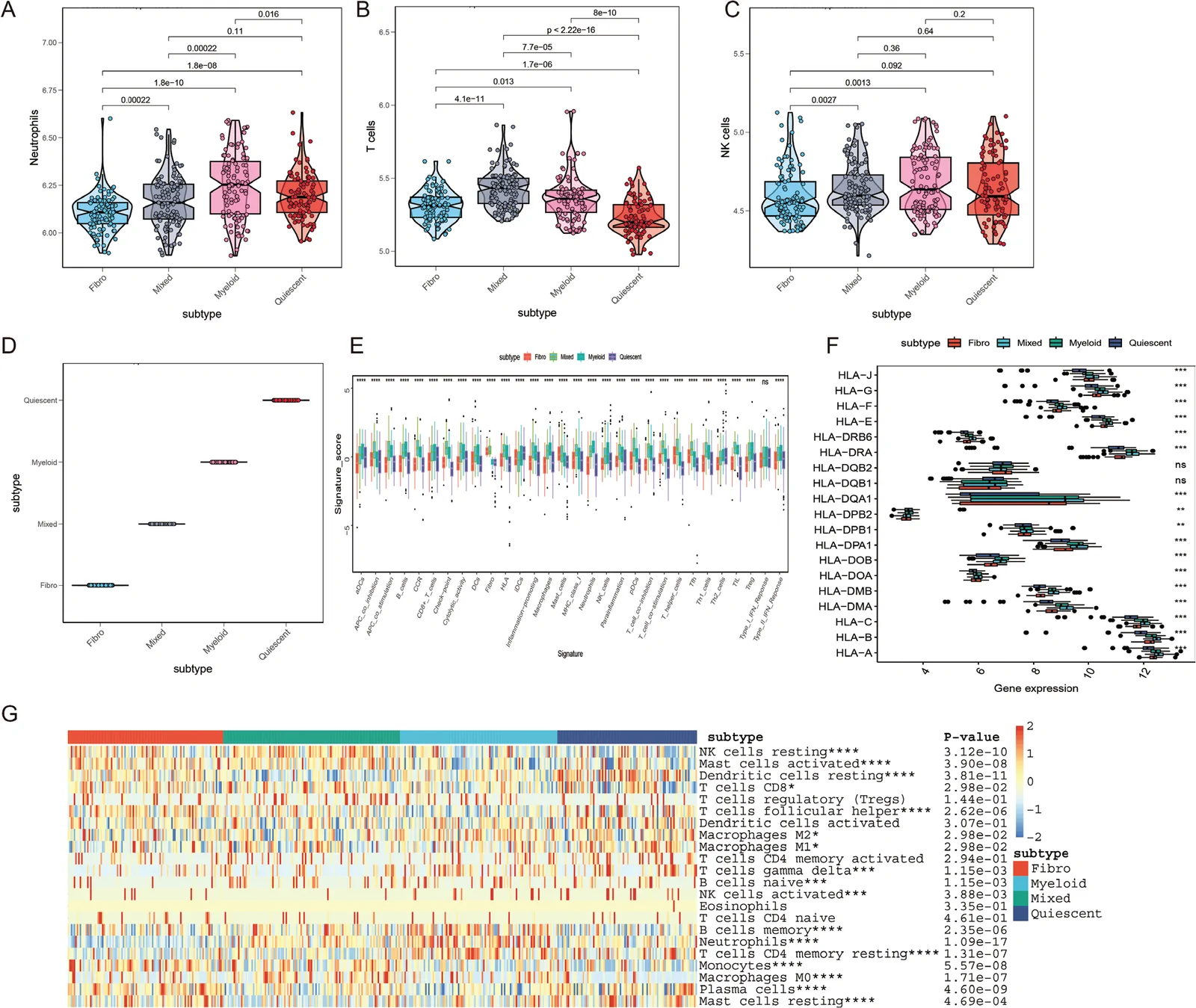
Immune microenvironment characterization and functional differences across periodontal disease subtypes. (A–C) Violin plots of immune cell fractions in the four periodontal disease subtypes (fibro, myeloid, mixed, and quiescent) based on MCPcounter and CIBERSORT algorithms. (A) Neutrophil content is significantly higher in the myeloid and mixed groups compared to other subtypes. (B) The abundance of T cells is more elevated in the mixed group and significantly differs from other subtypes. (C) NK cell content also shows significant enrichment in the myeloid and mixed groups (*p*-values shown for statistical comparisons). (D) PCA plot illustrating the segregation of samples based on subtype-specific immune profiles. The mixed and myeloid groups show a distinct clustering indicating inflammatory microenvironmental variations. (E) Boxplots of ssGSEA scores across 29 immune-related functional pathways underline that mixed and myeloid subtypes have more active immune functions and higher infiltration of immune cells compared to fibro and quiescent groups. (F) Expression variability of HLA-related genes across subtypes. The mixed and myeloid subtypes exhibit significantly higher HLA gene expression compared to the fibro and quiescent subtypes, indicating stronger antigen presentation and immune activation in these subtypes. (G) Heatmap showing immune cell signatures across all periodontal subtypes. Mixed and myeloid subtypes are associated with increased activated immune cells, such as Tregs, macrophages, and CD4+ T cells, reflecting a more inflamed immune microenvironment. Statistical significance: *p < 0.05, **p < 0.01, ***p < 0.001.

Assessment of the immune microenvironment using two independent algorithms, MCPcounter and CIBERSORT, indicated a significantly higher abundance of immune cells in the mixed and myeloid-dominant subtypes. These results were consistent with ssGSEA analysis, which also revealed increased immune cell content and enhanced immune function in these two subtypes (Figure 5A–E, G). Furthermore, HLA molecule expression was significantly elevated in both the mixed and myeloid-dominant subtypes (Figure 5F). The four identified periodontitis subtypes displayed distinct biological characteristics, suggesting a potential correlation with varying disease trajectories. The mixed and myeloid-dominant subtypes, in particular, appear to be characterized by a more pronounced inflammatory microenvironment.

### Identify key genes

**Figure 6:**
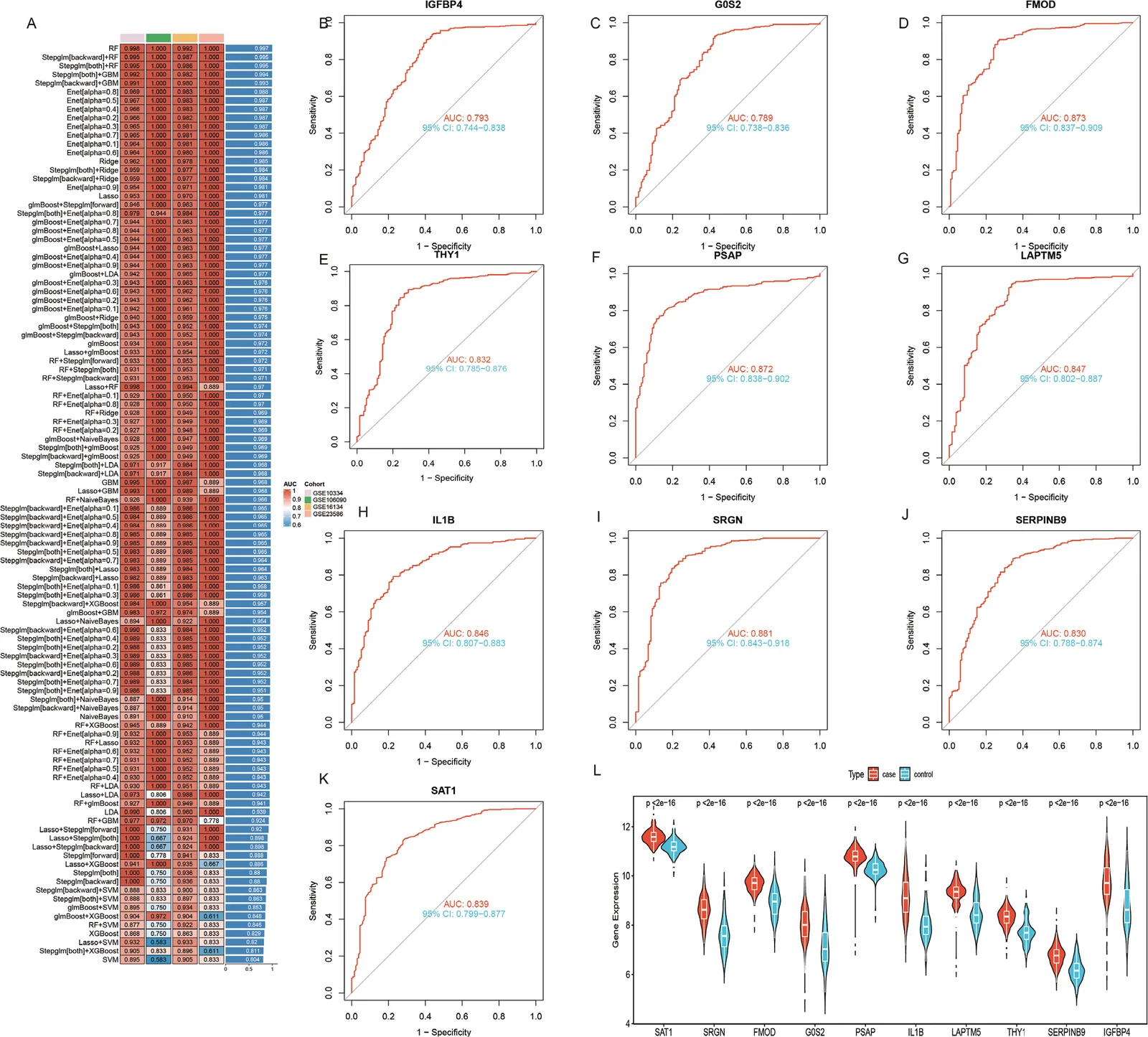
Diagnostic gene identification and model performance for periodontal disease. (A) Heatmap summarizing the diagnostic model performance across 106 machine learning algorithm combinations. RF achieved the highest average AUC values in all datasets, and 10 key diagnostic genes were filtered and identified: SAT1, SRGN, FMOD, G0S2, PSAP, IL1B, LAPTM5, THY1, SERPINB9, and IGFBP4. (B–K) Receiver operating characteristic (ROC) curves for each key diagnostic gene, demonstrating their performance in distinguishing periodontal disease tissues from healthy tissues. AUC (area under the curve) values are labeled for each gene, showcasing strong diagnostic utility (AUC > 0.7 for all genes). (L) Violin plots displaying the differential expression of the 10 diagnostic genes between periodontal disease samples (red) and healthy controls (blue). All genes show significantly higher expression in disease tissues (*p* < 0.05), validating their relevance to periodontal disease. Significance levels: *p < 0.05, **p < 0.01, ***p < 0.001.

To develop a robust diagnostic model for periodontitis, we compared the performance of several machine learning algorithms. A random forest (RF) model consistently outperformed others, achieving the highest average AUC across all datasets after feature filtering. This superior performance led to the identification of 10 genes (SAT1, SRGN, FMOD, G0S2, PSAP, IL1B, LAPTM5, THY1, SERPINB9, IGFBP4) as key diagnostic biomarkers (Figure 6A). Differential expression analysis (Figure 6L) and ROC curve analysis (Figure 6B–K) confirmed the significantly higher expression of these genes in periodontitis tissues and their ability to effectively discriminate between diseased and healthy tissues.

### Identification of hub genes via Mendelian randomization

To investigate the potential causal relationship between the candidate diagnostic biomarkers and periodontitis, we conducted an MR analysis using summary-data-based MR (SMR). From the 10 key diagnostic genes identified by the machine learning model (Figure 6A), six (G0S2, IGFBP4, IL1B, LAPTM5, PSAP, and SRGN) with qualified genetic instruments (eQTLs) in the GTEx v8 database were selected for causal inference.

**Figure 7:**
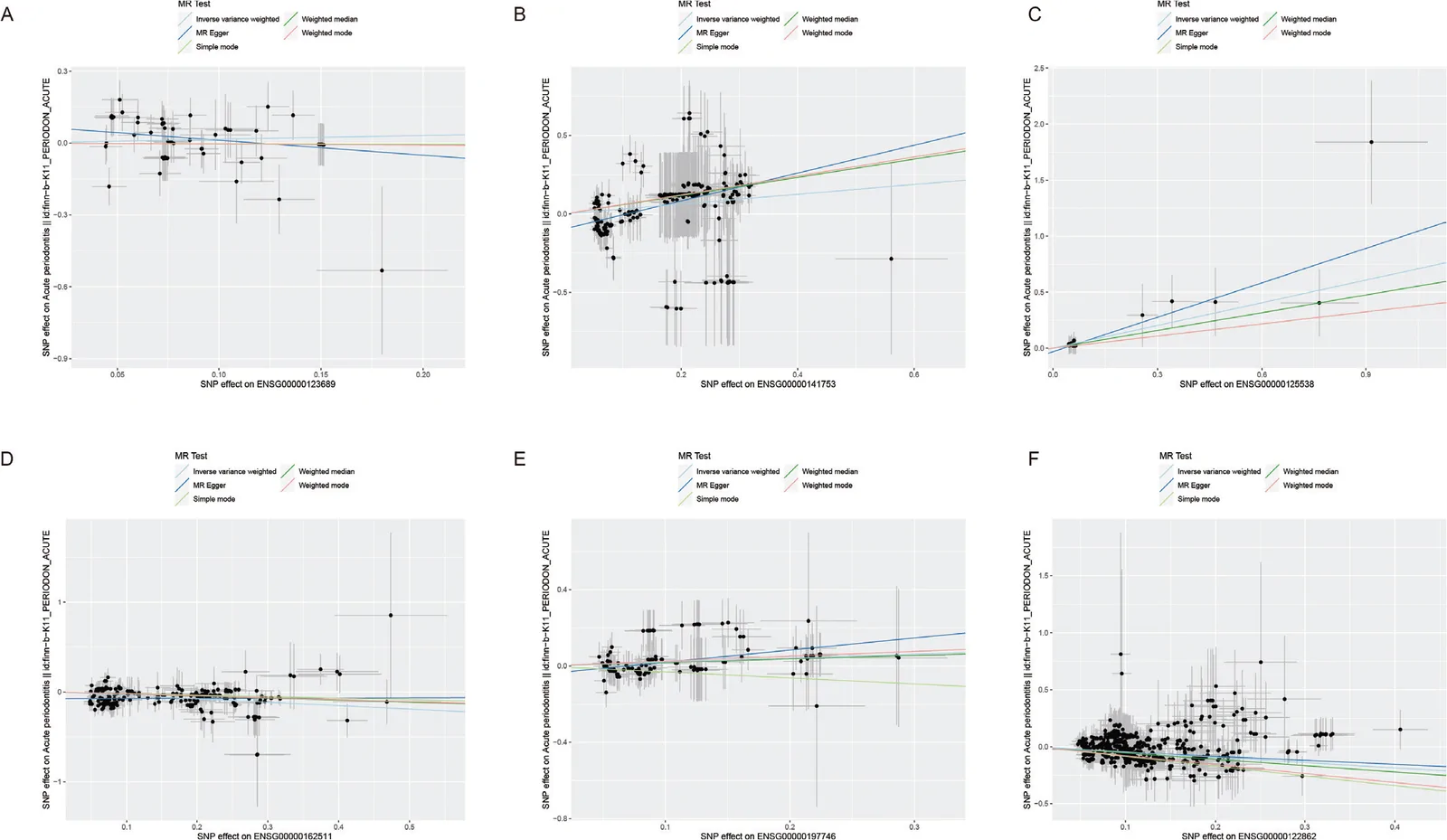
Mendelian randomization (MR) analysis of target genes associated with periodontal disease. (A–F) Scatter plots demonstrating the MR analysis results of six target genes: (A) G0S2, (B) IGFBP4, (C) IL1B, (D) LAPTM5, (E) PSAP, and (F) SRGN. Each plot shows SNP effects on gene expression levels (x-axis) versus SNP effects on periodontal disease outcomes (y-axis). The lines represent different MR methods: inverse variance weighted (IVW), weighted median, MR-Egger, and weighted mode.

We downloaded gene eQTL data for these genes from the SMR database and obtained periodontitis phenotype data from the finn-b-K11_PERIODON_ACUTE database within the IEU OpenGWAS project. The eQTL and outcome data were matched by SNP ID, subjected to quality control, and then analyzed using the SMR tool. IVW MR analysis revealed significant associations (*p* < 0.05) between periodontitis and five candidate genes: IGFBP4, IL1B, LAPTM5, PSAP, and SRGN (Figure 7A–F). In contrast, no significant association was observed for G0S2 (*p* > 0.05).

The results of the MR analysis suggest that IGFBP4, IL1B, LAPTM5, PSAP, and SRGN may play a role in periodontitis development, while G0S2 does not appear to be causally involved.

### Gene expression differences

**Figure 8:**
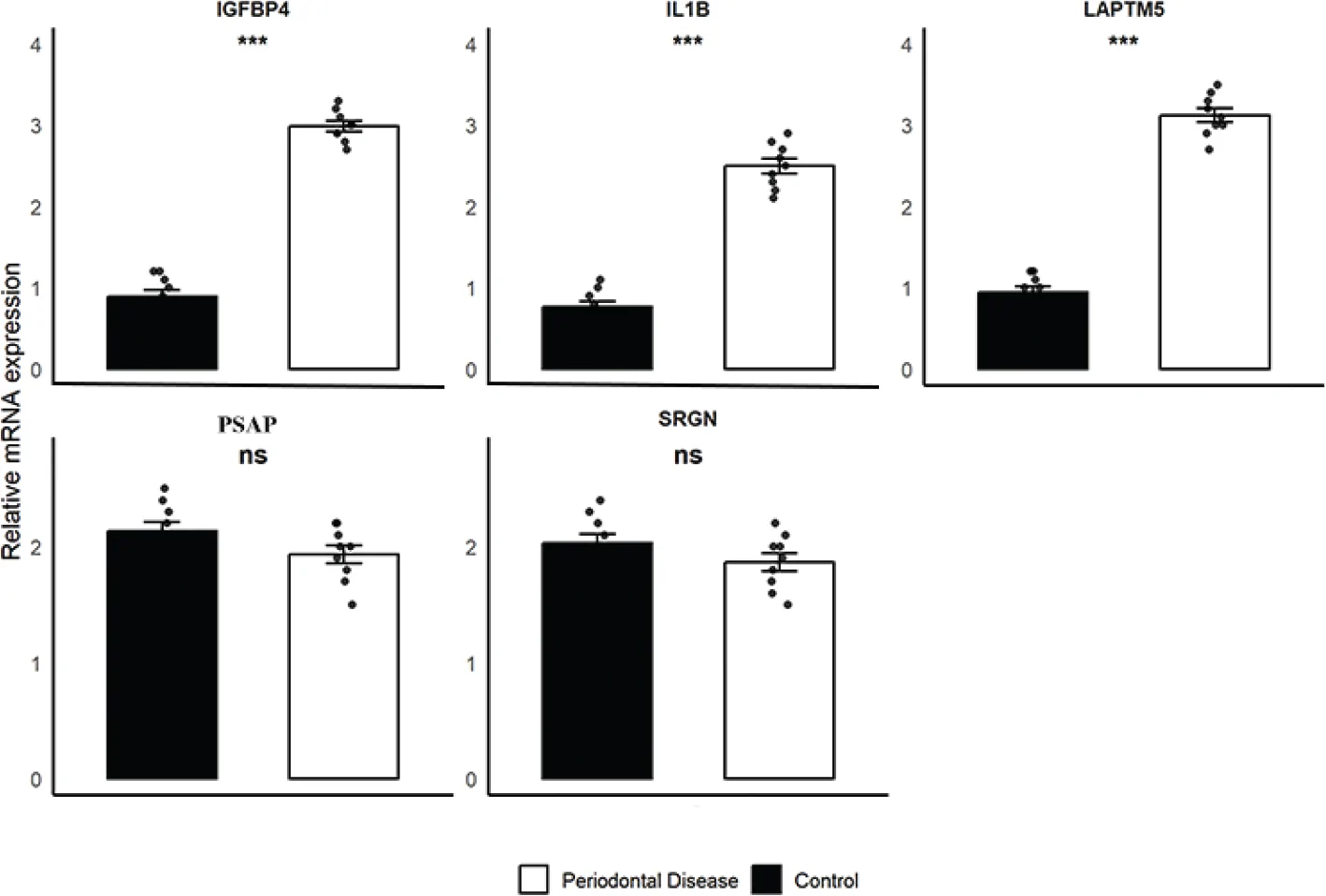
Differential expression of target genes in periodontal tissues between periodontitis patients and healthy controls. Relative mRNA expression levels of SRGN, PSAP, LAPTM5, IGFBP4, and IL1B were analyzed in gingival tissues obtained from patients with stage III/IV periodontitis (*n* = 13) and periodontally healthy controls (*n* = 15). Expression levels were quantified using RT-qPCR, with GAPDH serving as the internal reference gene. The mRNA expression levels of IGFBP4, IL1B, and LAPTM5 were significantly upregulated in the periodontitis group compared to the control group (***p < 0.001). The mRNA expression levels of SRGN and PSAP showed no significant differences between the periodontitis group and the control group (ns, not significant). Data are presented as mean ± SEM. Statistical analysis was performed using unpaired two-tailed Student’s t-test.

To further validate the results, we performed RT-qPCR analysis to assess the mRNA expression levels of IGFBP4, IL1B, LAPTM5, PSAP, and SRGN in gingival samples obtained from healthy individuals and periodontitis patients. Compared to healthy controls, we observed statistically significant differences in the expression of IGFBP4, IL1B, and LAPTM5 in periodontitis patients but not for PSAP and SRGN (Figure 8).

## Discussion

Periodontitis, a prevalent inflammatory disease caused by bacterial biofilms, is a leading cause of tooth loss in adults [24]. Its long history, evidenced by alveolar bone loss and tooth loss observed in ancient human remains [25], highlights its enduring impact on human health. Often, the subtle and initially mild symptoms of early-stage periodontitis lead to delays in seeking treatment, resulting in significant alveolar bone loss and rendering many teeth unsalvageable by the time patients present with noticeable tooth mobility [26]. The current clinical paradigm for the diagnosis of periodontitis relies on a combination of clinical parameters, namely PD and BOP and radiographic assessment of alveolar bone resorption. This information is then used to inform the subsequent treatment strategy. The link between oral health and systemic disease has been a subject of increasing interest since W.D. Miller’s 1891 assertion that the mouth serves as a focus of infection. Over the past two decades, rigorous epidemiological studies and case–control research, guided by principles of evidence-based medicine, have established associations between periodontitis and various systemic conditions. For example, Tonetti et al. demonstrated a potential link between periodontal pathogens and atherosclerosis, suggesting that bacterial products might disseminate hematogenously to affect vascular health [27]. The systemic effects of periodontitis, including inflammatory responses [28] and adverse pregnancy outcomes [29], underscore the disease’s profound impact beyond the oral cavity. However, current diagnostic and treatment methods often fail to detect the disease in its early stages. This critical gap highlights the urgent need for early diagnostic biomarkers to improve patient management and outcomes.

The advent of single-cell RNA sequencing (scRNA-seq) has significantly advanced the study of complex biological systems, particularly in the context of disease. This technology facilitates a comprehensive analysis of cellular heterogeneity within disease microenvironments by providing a high-throughput method for profiling the transcriptome of individual cells. This allows for the identification and characterization of rare cell populations and provides a deeper understanding of their functional roles in both physiological and pathological conditions [30]. Using scRNA-seq, we characterized the complex cellular interactions and signaling pathways involved in periodontal disease progression. Analysis of a single-cell dataset from periodontitis patients revealed distinct characteristics of various cell types and their communication patterns. This analysis highlighted the importance of intercellular communication in periodontal disease pathogenesis, revealing intricate interactions between immune cells, fibroblasts, and other cell populations. Moreover, employing machine learning techniques, we identified novel molecular subtypes of periodontitis and key genes associated with disease development.

Interleukin-1β (IL-1β) is a pro-inflammatory cytokine with a central role in the pathogenesis of systemic inflammatory diseases. Its potent pro-inflammatory activity is mediated by its secretion from immune cells, such as monocytes and macrophages, triggering a cascade of inflammatory event [31, 32]. IL-1β initiates and amplifies inflammatory responses through a complex signaling mechanism. This mechanism is triggered by the binding of IL-1β to its specific receptor, leading to the activation of key signaling pathways, most notably the NF-κB and MAPK pathways [33]. L-1β contributes significantly to the pathogenesis of rheumatoid arthritis (RA). By promoting synovial cell proliferation and the production of inflammatory enzymes (Matrix metalloproteinases (MMPs) and (Cyclooxygenase‑2) COX-2), IL-1β drives synovial inflammation and joint damage, resulting in local cartilage and bone erosion and systemic inflammatory responses [34]. IL-1β plays a significant role in the progression of atherosclerosis. Its pro-inflammatory effects include the activation of endothelial cells, leading to increased vascular permeability and enhanced monocyte adhesion to the endothelium. These monocytes subsequently differentiate into foam cells, contributing to plaque formation. In addition, IL-1β stimulates the proliferation and migration of vascular smooth muscle cells, further promoting plaque growth. The release of pro-inflammatory mediators by IL-1β further accelerates this process, culminating in vascular stenosis and arteriosclerosis [35]. IL-1β, a prominent early biomarker for periodontitis, plays a multifaceted role in disease progression. Its pro-inflammatory actions include increasing adhesion molecule expression, promoting leukocyte recruitment, stimulating the production of inflammatory mediators and MMPs, and activating immune cells. Furthermore, IL-1β stimulates osteoclastogenesis while inducing apoptosis of cells responsible for extracellular matrix production, thus impairing tissue repair [36]. This pivotal role is further highlighted by its involvement in the transition from gingivitis to irreversible periodontitis [37], possibly in concert with Receptor activator of nuclear factor‑κB ligand (RANKL) in alveolar bone loss [38]. The maturation and secretion of IL-1β are critically dependent on the inflammasome, a multiprotein complex [39]. Various inflammasomes, including those from the NOD‑like receptors (NLR) family such as NLRP1, NLRP2, NLRP3, and AIM2, are involved in this process. Among these, NLRP3 is the most well-understood [40, 41]. Elevated NLRP3 expression has been observed in gingival tissue and saliva of individuals with periodontitis [42], indicating a strong association between inflammasome activation and increased IL-1β production. Furthermore, the efficacy of therapies targeting NLRP3 inflammasome inhibition in experimental periodontitis models [43–45] highlights the potential of this pathway as a therapeutic target and provides valuable insights for future research efforts.

IGFBP4 belongs to the family of seven IGF-binding proteins (IGFBPs) and was initially understood to primarily regulate the bioavailability of insulin-like growth factors (IGFs) in the circulatory system, acting as a bidirectional modulator of IGF function in response to environmental changes [46]. However, a growing body of evidence reveals a much broader functional scope for IGFBP4. Beyond its IGF-dependent effects, IGFBP4 has been shown to directly influence transcriptional regulation, cell migration, and apoptosis, highlighting its significant involvement in various physiological and pathological contexts [46]. Research suggests that IGFBP4, as part of the IGF-binding protein family, contributes to the development and progression of various tumors. Its expression levels may therefore serve as a valuable prognostic indicator for different types of cancer [47]. Synovial fluid from RA patients shows significantly higher IGFBP4 levels compared to that of healthy individuals [48]. A study of Malassez cells revealed the presence of only IGFBP4 and IGFBP6 [49]. Analysis of IGF components in human permanent teeth revealed the presence of IGFBP4 within the periodontal ligament; however, IGFBP4 was not detected in the cementum or pulp tissues [50]. Previous research showed that high concentrations of pomegranate peel extract significantly increased IGFBP4 expression in periodontal tissues, promoting tissue repair. In contrast, our study found significantly higher IGFBP4 expression in gingival tissues from patients with periodontitis compared to healthy controls. This elevated expression may represent a compensatory response to tissue damage, but further research is needed to elucidate the underlying mechanisms and prognostic significance.

LAPTM5, a lysosomal transmembrane protein, shows preferential expression in hematopoietic cells. Although the integrity of the LAPTM5 protein is crucial for maintaining lysosomal stability, its overexpression can induce lysosomal cell death (LCD). In addition to this role, LAPTM5 is involved in the activation of autophagy and has been shown to be closely associated with the regulation of immune and inflammatory processes. Therefore, LAPTM5 regulates diverse physiological processes and is implicated in various diseases [51]. In a study of MC3T3-e1 mineralization, RUNX2 appeared to regulate LAPTM5 expression, resulting in a transient increase in LAPTM5-mediated autophagy. The concomitant increase in LAPTM5 expression during osteogenic mineralization suggests a role for the RUNX2/LAPTM5 axis in promoting both autophagy and osteogenesis [52]. Another study demonstrated that the lysosomal system is involved in both osteogenesis and osteoclastogenesis, with LAPTM5 identified as a key protein in this process [53]. The progression of periodontal disease is characterized by the destruction and remodeling of alveolar bone. Investigating the roles of LAPTM5 in both osteogenesis (bone formation) and osteoclastogenesis (bone resorption) may provide crucial insights into the mechanisms underlying bone loss in periodontitis, potentially leading to the development of improved diagnostic and therapeutic strategies.

This study systematically characterized the periodontal disease microenvironment at the molecular level by integrating single-cell RNA sequencing and MR analysis, identifying key biomarkers and validating them experimentally. Four novel molecular subtypes of periodontitis were identified: fibroblast, myeloid, fibroblast/myeloid quiescent, and fibroblast/myeloid mixed. These subtypes exhibited distinct biological patterns, with the mixed and myeloid subtypes potentially exhibiting a stronger inflammatory microenvironment. This provides crucial insights into the pathophysiology of periodontitis. Furthermore, using multiple machine learning models, we identified key genes strongly associated with periodontitis, including IGFBP4, IL1B, and LAPTM5, and validated their differential expression via RT-qPCR.

Several limitations require further optimization. First, the relatively small sample size may limit the generalizability of the findings, particularly the RT-qPCR validation stage which included only 13 periodontitis patients, potentially hindering a comprehensive reflection of molecular characteristics in a larger population. Second, the study focused primarily on gene expression, neglecting the investigation of protein-level regulatory mechanisms, which exert more direct effects on disease pathogenesis. Third, the study relied primarily on publicly available datasets and experimental validation, lacking longitudinal patient follow-up data to observe the dynamic changes in key gene expression throughout disease progression.

Future studies could utilize animal models to investigate the dynamic changes in key genes during disease pathogenesis and treatment, further validating their clinical value as diagnostic biomarkers and therapeutic targets. These comprehensive investigations will provide a stronger theoretical foundation and practical guidance for personalized and precision medicine approaches to periodontitis.

## Conclusion

In conclusion, our investigation has identified four distinct periodontitis subtypes (fibroblast-dominant, myeloid-dominant, fibroblast/myeloid quiescent, and fibroblast/myeloid mixed), along with three key diagnostic biomarkers (IGFBP4, IL1B, and LAPTM5), as potential therapeutic targets for medical intervention in periodontitis. This genetic and molecular evidence suggests that targeting these subtypes and biomarkers could have significant clinical benefits, informing the development of precise diagnostic tools and targeted therapies for periodontitis management.

## Supplementary Material


