# Supplementary material for: Integrating machine learning and multi-omics analysis to identify and validate key genes associated with periodontitis
Source: Acta Odontol Scand. 2026 Aug 19;85:46662. doi: 10.2340/aos.v85.46662 (PMC13494742; doi:10.2340/aos.v85.46662)
Supplement: Supplementary file 1 [file AOS-85-46662-s1.pdf]

Supplementary material has been published as submitted. It has not been copyedited or typeset by Acta Odontologica Scandinavica.

Supplementary table 1

Primer Information	Primer Name	Primer Sequence (5'-3')	Fragment Length (bp)	Annealing Temperature（℃）
NM_002046	H-GAPDH-S	GGAAGCTTGTCATCAATGGAAATC	168	60
	H-GAPDH-A	TGATGACCCTTTTGGCTCCC		60
NM_001552.3	H-igfbp4 -S	GGCAAGATGAAGGTCAATGGG	182	60
		TGCTTGGGGTGGAAGTTGC		60
NM_000576.3	H-IL1B(2)-S	TACCTGTCCTGCGTGTTGAAA	171	60
	H-IL1B(2)-A	GGTGCTGATGTACCAGTTGGG		60
NM_006762.3	H-LAPTM5-S	CAATGTCCGCATCGCAACC	174	60
	H-LAPTM5-A	AGCATGGTGATGAGCAGGAA		60
NM_001871.3	H-CPB1(1)-S	ATGAAACTTACTGTGGACCTGCC	176	60
PASP	H-CPB1(1)-A	CAACTCAGCATTGTTCTCACCG		60
NM_001321053.2	H-SRGN-S	TCCAACAAGATCCCCCGTCT	169	60
	H-SRGN-A	AATCCTGTTCCATTTCCGTTAGG		60
